# Dimerization facilitates the conformational transitions for bacterial phosphotransferase enzyme I autophosphorylation in an allosteric manner

**DOI:** 10.1002/2211-5463.12260

**Published:** 2017-07-17

**Authors:** Ko On Lee, Young‐Joo Yun, Iktae Kim, Jeong‐Yong Suh

**Affiliations:** ^1^ Department of Agricultural Biotechnology and Research Institute of Agriculture and Life Sciences Seoul National University Korea; ^2^ Institute for Biomedical Sciences Shinshu University Nagano Japan

**Keywords:** allostery, conformational transition, dimerization, phosphotransferase system

## Abstract

The bacterial phosphotransferase system is central to sugar uptake and phosphorylation. Enzyme I (EI), the first enzyme of the system, autophosphorylates as a dimer using phosphoenolpyruvate (PEP), but it is not clearly understood how dimerization activates the enzyme activity. Here, we show that EI dimerization is important for proper conformational transitions and the domain association required for the autophosphorylation. EI(G356S) with reduced dimerization affinity and lower autophosphorylation activity revealed that significantly hindered conformational transitions are required for the phosphoryl transfer reaction. The G356S mutation does not change the binding affinity for PEP, but perturbs the domain association accompanying large interdomain motions that bring the active site His189 close to PEP. The interface for the domain association is separate from the dimerization interface, demonstrating that dimerization can prime the conformational change in an allosteric manner.

**Abbreviations**
EI: enzyme I
EIC: C‐terminal domain of enzyme I
EIN: N‐terminal domain of enzyme I
PEP: phosphoenolpyruvate

Enzyme I (EI) is the first protein of the bacterial phosphotransferase system, which catalyzes the sugar transport and phosphorylation 1, 2. EI catalyzes an Mg^2+^‐dependent autophosphorylation reaction using phosphoenolpyruvate (PEP) as a substrate, and a phosphoryl transfer reaction to histidine‐containing phosphocarrier protein, HPr. EI consists of an N‐terminal domain (EIN) comprising an HPr binding subdomain (EINα), and a catalytic phosphohistidine subdomain (EINαβ), and a C‐terminal dimerization domain (EIC) for PEP binding 3, 4. EI switches between open and closed conformational states via large domain motions for its autophosphorylation reaction. Free EI adopts an open state that is relevant to a phosphoryl transfer reaction between EI and HPr 5, 6, and PEP binding to EI induces a closed state that enables the autophosphorylation reaction 7. Transition between two conformational states involves a hinge motion of EINα and a swivel motion of EINαβ (Fig. 1). Once the hinge motion disengages EINα from EINαβ, the active site His189 of EINαβ can be brought to PEP bound on EIC without steric clash by the swivel motion.

**Figure 1:**
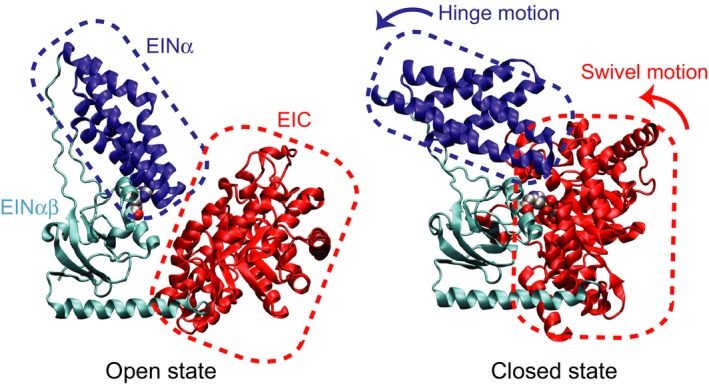
The open (PDB code 2kx9) and closed (PDB code 2hwg) conformations of EI shown in a cartoon diagram. The EINα subdomain is colored in blue, the EINαβ subdomain in cyan, and the EIC domain in red. The active site His189 in the open conformation and phospho‐His189 and oxalate in the closed conformation are shown as space‐filling models. EINα and EIC are highlighted with dashed rectangles in blue and red, respectively, to visualize the hinge and swivel domain motions during the open‐to‐closed conformational switch of EI upon PEP binding.

EI forms a dimer via EIC, and the dimerization of EI and isolated EIC has been extensively investigated by sedimentation equilibrium experiments 8, 9. PEP binding largely increased EI dimerization, inducing a compact EI dimer formation. It is known that an EI dimer is capable of the autophosphorylation reaction, but how the dimerization makes EI functionally competent is not clearly understood 10, 11. Previously, EI with a defect in dimerization and autophosphorylation activity was found in *Salmonella typhimurium* strains, and a G356S mutation was linked to the reduced EI activity 12, 13. When the G356S mutation was introduced to EI of *Escherichia coli*, EI(G356S) also exhibited significantly weaker dimerization and reduced autophosphorylation activity 13. Here, we used EI(G356S) of *E. coli* to address the mechanistic link between EI dimerization and autophosphorylation activity. We demonstrate that EI dimerization facilitates the conformational transitions between open and closed states required for the autophosphorylation reaction.

## Materials and methods

### Cloning, protein expression, and purification

Full‐length and domain deletion mutants of EI(1–575) were cloned into a pET11 or a pET15b vector with an N‐terminal His_6_ tag. EI_A_ denotes the active site H189A mutation in the αβ subdomain of EI, and EIC(G356S) denotes the G356S mutation in EIC(231–575). EINαβ(1–21, 146–249) was cloned to remove EINα(25–143) and loop regions that connect EINαβ and EINα from EIN(1–249). We note that EIN and EIC are linked by a long connecting α‐helix(231–260) that is partly included in the EIN and EIC domain constructs in this study for optimal protein stability. Thus, EI_A_^ΔEINα^(G356S) denotes the EI(1–21, 146–575, H189A, G356S) mutant. The plasmids were introduced into *E. coli* strain BL21star (DE3) (Novagen, Madison, WI, USA), and the transformant was grown in either Luria–Bertani or minimal media with ^15^NH_4_Cl as the sole nitrogen source. The culture was induced at an A_600_ of ~ 0.8 by the addition of 1 mm isopropyl‐β‐d‐thiogalactopyranoside, and harvested by centrifugation after 4 h of induction. The cell pellet was resuspended in 50 mL (per liter of culture) of 50 mm Tris/HCl, pH 7.4, 200 mm NaCl, 2 mm β‐mercaptoethanol, 1 mm phenylmethylsulfonyl fluoride, and 1 tablet of protease cocktail inhibitor (S8830 SIGMAFAST; Sigma‐Aldrich, St. Louis, MO, USA). The suspension was lysed by three passages through Emulsiflex (Avestin, Ottawa, ON, Canada) after homogenizing and was centrifuged at 24 000 ***g*** for 20 min at 4 °C. The supernatant fraction was filtered and loaded onto a DEAE column or a HisTrap column (GE Healthcare, Chicago, IL, USA). The fractions containing the proteins were purified by a Superdex200 column or a Superdex75 column (GE Healthcare) equilibrated with 20 mm Tris/HCl, pH 7.4, 200 mm NaCl, and 2 mm β‐mercaptoethanol and were then further purified by monoQ anion‐exchange column (8 mL; GE Healthcare) with a 160‐mL gradient of 1 m NaCl. All proteins were dialyzed against 20 mm Tris/HCl, pH 7.4, 100 mm NaCl, 2 mm β‐mercaptoethanol, and 4 mm MgCl_2_ (buffer A) for further analysis.

### Circular dichroism

Circular dichroism (CD) spectroscopy was conducted at 25 °C using a Chirascan™‐plus CD spectrometer. Wave scans were acquired by sampling data at 1‐nm intervals between 200 and 250 nm for far UV CD measurement. Far UV CD spectroscopy was carried out with 10 μm of proteins in buffer A using a 0.5‐mm quartz cuvette. Each far UV CD spectrum was obtained from an average of three scans, and the results were presented as mean residue ellipticity (deg cm^2^·dmol^−1^) at each wavelength.

### Multiangle light scattering

Purified proteins were characterized by multiangle light scattering (MALS) following the size exclusion chromatography. Two hundred micromolar proteins was injected onto a WTC‐0303 column (Wyatt Technology, Santa Barbara, CA, USA) equilibrated with buffer A in the presence and absence of 10 mm PEP. The chromatography system was connected to an 18‐angle light scattering detector (DAWN HELEOS II; Wyatt Technology), a dynamic light scattering detector (DynaPro Nanostar; Wyatt Technology), and a refractive index detector (Optilab t‐rEX; Wyatt Technology). Data were collected every 1 s at a flow rate of 0.5 mL·min^−1^ at 25 °C. Data analysis was carried out using the software package astra 6 (Wyatt Technology) to determine the molar mass and mass distribution of the sample.

### Isothermal titration calorimetry

Isothermal titration calorimetry (ITC) experiments were performed using the ITC_200_ microcalorimeter (Malvern) at 25 °C. Three hundred micromolar PEP was titrated into 30 μm EI_A_(G356S) or EI_A_^ΔEINα^(G356S) in buffer A, and 3 mm PEP was titrated into 300 μm EIC(G356S). Twenty consecutive 2‐μL aliquots of PEP were titrated into the proteins in the cell. The duration of each injection was 4 s, and injections were made at intervals of 150 s. The heats associated with the dilution of PEP were subtracted from the measured heats of binding. ITC titration data were analyzed with the origin version 7.0 program (Northampton, MA, USA) provided with the instrument.

### NMR spectroscopy

NMR spectra were recorded at 25 °C on Bruker Avance 600‐ and 900‐MHz spectrometers equipped with *x*,* y*,* z*‐shielded or *z*‐shielded gradient triple resonance probes. To examine the binding interface of isolated EINαβ for isolated EIC(G356S), two‐dimensional ^1^H‐^15^N heteronuclear single quantum correlation spectra of 0.3 mm ^15^N‐labeled EINαβ(H189A) were obtained titrating with unlabeled EIC(G356S) in the presence and absence of 10 mm PEP in buffer A. The backbone chemical shifts of EINαβ(H189A) were obtained by the comparison with previously assigned chemical shifts of EIN, and confirmed by three‐dimensional triple resonance through‐bond scalar correlation CBCACONH and HNCACB experiments. NMR spectra were processed using the NMRPipe 14 program and analyzed using the PIPP 15 and the NMRView 16 programs.

## Results and Discussion

### Impact of G356S mutation on EI and EIC dimerization

**Figure 2:**
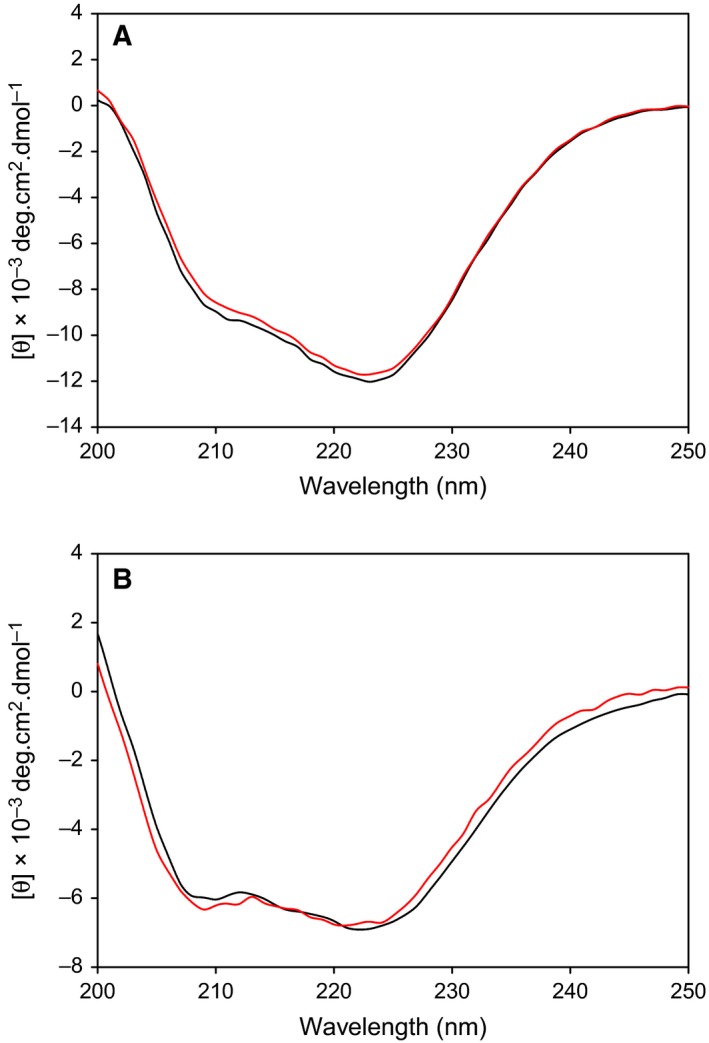
Far UV (200–250 nm) circular dichroism spectra of (A) EI_A_ and EI_A_(G356S) in black and red, respectively, and (B) EIC and EIC(G356S) in black and red, respectively.

**Figure 3:**
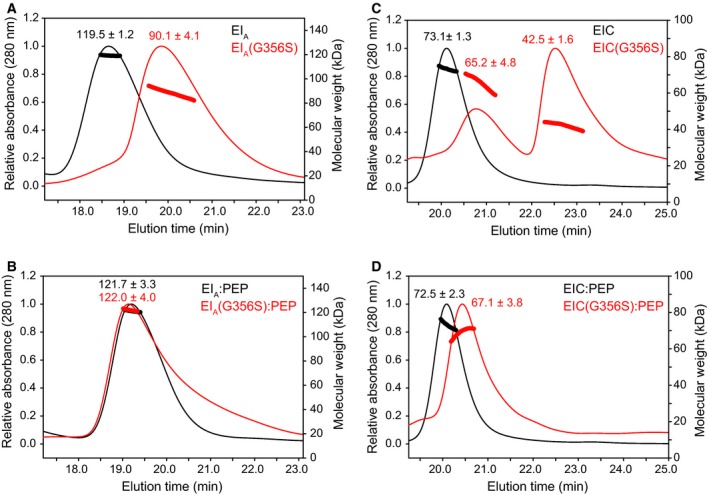
Multiangle light scattering analysis of EI_A_ in black and EI_A_(G356S) in red (A) in the absence of PEP and (B) in the presence of PEP, and multiangle light scattering analysis of EIC in black and EIC(G356S) in red (C) in the absence of PEP, and (D) in the presence of PEP. The absolute molecular masses obtained from the light scattering data are shown above individual elution peaks.

We employed the active site mutant EI_A_ (His189 replaced with Ala) in this study, so that PEP binding to EI would not proceed with the autophosphorylation reaction. The circular dichroism spectra were very similar between EI_A_ and EI_A_(G356S), and also between EIC and EIC(G356S), indicating that the G356S mutation did not perturb the secondary structures (Fig. 2). We examined the dimerization states of the mutants using MALS data. EI_A_ (~ 63.5 kDa) exhibited 119.5 ± 1.2 kDa at 50 μm of the elution concentration, indicating ~ 90% of a dimer (Fig. 3A). The elution concentration was obtained from the UV_280_ absorption at the peak height. On the other hand, EI_A_(G356S) exhibited 90.1 ± 4.1 kDa at 50 μm of the elution concentration, indicating ~ 40% of a dimer (Fig. 3A). The estimated equilibrium dissociation constant (*K*_D_) of an EI_A_ dimer from the dimer fraction was ~ 0.4 μm that was in good agreement with *K*_D_ ~ 0.6 μm from the sedimentation velocity data of wild‐type EI 5. The *K*_D_ value of EI_A_(G356S) was calculated as ~ 30 μm, indicating a ~ 75‐fold reduction in the dimerization affinity. PEP binding, however, largely restored the EI dimerization, and both EI_A_ and EI_A_(G356S) formed predominantly a dimer (> 90%) upon PEP binding (Fig. 3B).

EIC (~ 38.5 kDa) exhibited 73.1 ± 1.3 kDa at 50 μm of the elution concentration, and mostly existed as a dimer (Fig. 3C), which was consistent with *K*_D_ ~ 6 nm from sedimentation velocity data 9. EIC(G356S) eluted as two peaks of 65.2 ± 4.8 kDa and 42.5 ± 1.6 kDa with a 1 : 2 ratio, both of which corresponded to a mixture of a monomer and a dimer (Fig. 3C). The separate elution of two peaks suggests a possible conformational heterogeneity of EIC(G356S), which was not evident in EI_A_(G356S). The lower bound of *K*_D_ value of EIC(G356S) was estimated as 5 μm from the higher molecular weight fraction, indicating that the G356S mutation caused a significantly larger impact on the dimerization of isolated EIC than that of EI. PEP binding increased the dimerization of EIC(G356S) such that EIC(G356S):PEP appeared as a single peak of 67.1 ± 3.8 kDa, indicating ~ 80% of a dimer (Fig. 3D). Taken together, the G356S mutation considerably reduced the dimerization of both EI_A_(G356S) and EIC(G356S), but PEP binding largely restored the dimerization affinity, especially for EI_A_(G356S). Although the estimated dimerization constants from the MALS data are only semiquantitative, they are consistent with the reported values of EI_A_ and EIC from previous analytical centrifugation experiments 5, 9. As EI_A_(G356S) formed a tight dimer that was comparable to EI_A_, we examined the impact of G356S mutation on the conformational transitions of EI during the phosphoryl transfer reaction.

### Impact of G356S mutation on PEP binding and conformational transitions of EI

It has been reported that EI(G356S) suffers from significantly reduced autophosphorylation activity with only ~ 4% of *V*_max_ of autophosphorylation compared to EI 13. As EI(G356S) formed a weaker dimer than EI, the reduced activity was supposed to originate from an EI(G356S) monomer. Our study, however, showed that EI(G356S) could form a tight dimer in the presence of PEP, suggesting that the reduced activity could be intrinsic to the EI(G356S) dimer. We investigated the impact of G356S mutation on PEP binding and conformational transitions of EI using EI_A_(G356S) and its domain deletion mutants by calorimetry and NMR spectroscopy. We recall that EI switches from an open state to a closed state upon PEP binding, which involves a hinge motion of EINα and an association of EINαβ and EIC by a swivel motion 5, 7. The apparent free energy (Δ*G*_app_) of PEP binding to EI_A_ is comprised of the free energy contributions from intrinsic PEP binding of EIC (Δ*G*_PEP_) and accompanying conformational transitions (Δ*G*_hinge_ and Δ*G*_asso_), such that Δ*G*_app_(EI_A_) = Δ*G*_PEP_ **+ Δ*G*_hinge_ + Δ*G*_asso_, as previously described 17. When the EINα subdomain is removed from EI_A_, EI_A_^ΔEINα^ binds to PEP and switches to the closed state without the hinge motion, yielding Δ*G*_app_(EI_A_^ΔEINα^) = Δ*G*_PEP_ **+ Δ*G*_asso_. The PEP binding (Δ*G*_PEP_) can be directly measured using isolated EIC that binds to PEP without domain motions 18. Linear combinations of measured Δ*G*_app_(EI_A_), Δ*G*_app_(EI_A_^ΔEINα^), and Δ*G*_PEP_ values can then provide individual Δ*G*_PEP_, Δ*G*_hinge_, and Δ*G*_asso_ of EI_A_. Recently, NMR residual dipolar coupling and small‐angle X‐ray scattering data have revealed that EI(H189A) forms a mixture of partially closed and closed states in the presence of PEP 19. The partially closed state represents the intermediate state between the open and closed states, where the domain orientation of EINα and EINαβ resembles the closed state conformation, but EINαβ is not fully engaged with EIC to catalyze the in‐line phosphoryl transfer reaction with PEP. Thus, it should be mentioned that Δ*G*_asso_ in our study includes the domain association to form the partially closed state in addition to the closed state observed in the crystal structure. Given that buried accessible surface in the closed state is 3.4 times larger than that in the partially closed state, we speculate a larger contribution of the closed state to the measured Δ*G*_asso_ 19.

**Figure 4:**
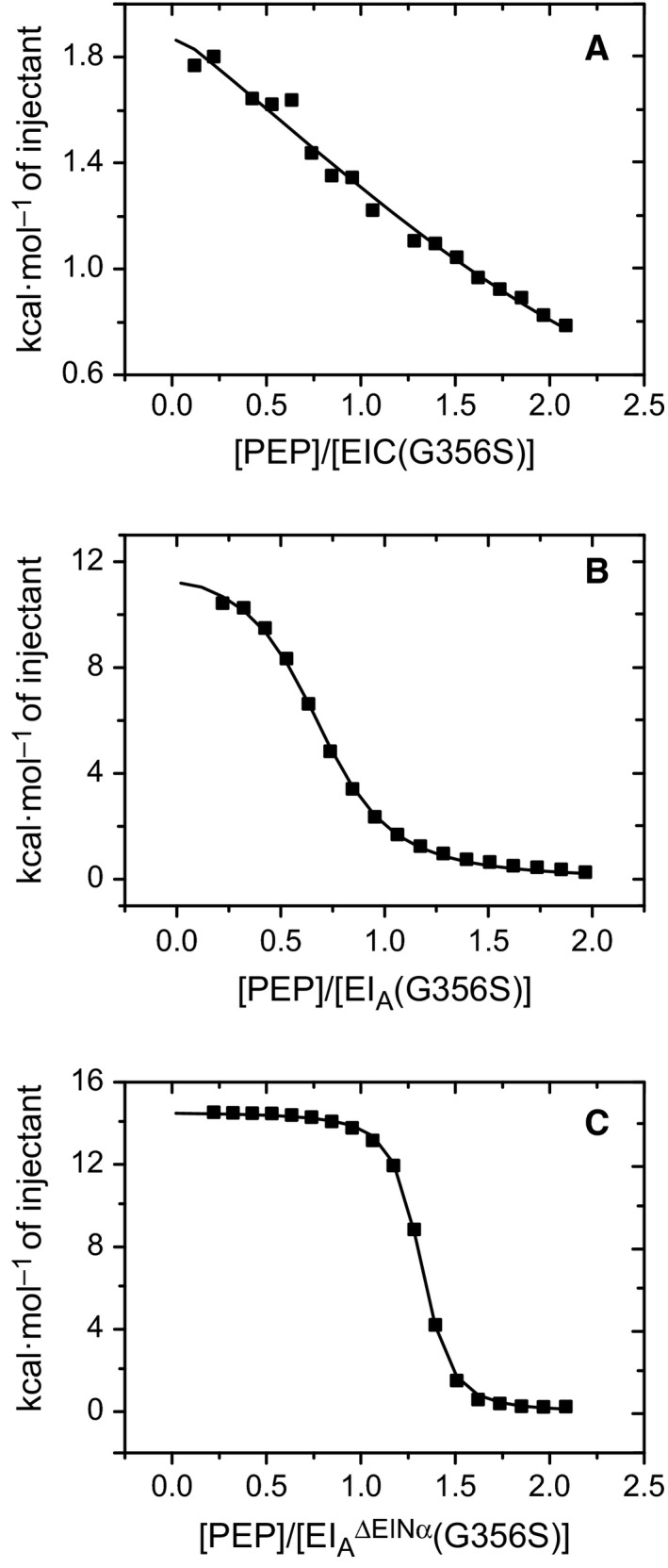
Integrated heats of injections from the titration between (A) PEP and EIC(G356S), (B) PEP and EI_A_(G356S), and (C) PEP and EI_A_^ΔEINα^(G356S). The squares are the experimental data, and the lines represent the least‐squares best‐fit curves derived for one set of sites binding model. The equilibrium dissociation constants and thermodynamic parameters are given in Table 1.

We first examined whether the G356S mutation would affect the intrinsic PEP binding of EIC. The *K*_D_ value of PEP binding to EIC(G356S) was measured as ~ 310 ± 100 μm (Fig. 4A), which was comparable to ~ 260 ± 80 μm obtained for EIC 18. Thus, G365S mutation did not perturb the intrinsic PEP binding affinity of EIC. We then examined the conformational transitions of EI_A_(G356S) upon PEP binding. The Δ*G*_app_ value for overall PEP binding and the conformational transitions of EI_A_(G356S) was measured as −6.6 ± 0.1 kcal·mol^−1^ (Fig. 4B), which was −2.1 kcal·mol^−1^ smaller than that of EI_A_ (Δ*G*_app_ **= −8.7 ± 0.2 kcal·mol^−1^). The decreased free energy change suggests that the conformational transitions of EI_A_(G356S) upon PEP binding were less efficient than EI_A_. We further examined whether the hinge motion of EINα (Δ*G*_hinge_) or the association between EINαβ and EIC (Δ*G*_asso_) by the swivel motion was affected by the G356S mutation (Fig. 4C). Δ*G*_hinge_ was obtained by subtracting Δ*G*_app_ (−8.0 ± 0.1 kcal·mol^−1^) of EI_A_^ΔEINα^(G356S) from Δ*G*_app_ (−6.6 ± 0.1 kcal·mol^−1^) of EI_A_(G356S). Δ*G*_hinge_ of EI_A_(G356S) was 1.4 ± 0.1 kcal·mol^−1^, which was largely the same as that of EI_A_ (Table 1). On the other hand, the G356S mutation significantly reduced the domain association between EINαβ and EIC. Δ*G*_asso_ of EI_A_(G356S) was −3.3 ± 0.2 kcal·mol^−1^, whereas that of EI_A_ was −5.2 ± 0.2 kcal·mol^−1^, revealing that the G356S mutation reduced the domain association by −1.9 ± 0.3 kcal·mol^−1^ (Table 1).

**Table 1:** Thermodynamic parameters for PEP binding and conformational transitions of EI mutants. ‘EI_A_ + PEP’ denotes the measured thermodynamic parameters for the binding between EI_A_ and PEP. Thermodynamic parameters for the conformational transition accompanying hinge and swivel motions were obtained by linear combinations of measured parameters as described in the main text

	*K*_D_ (μs)	▵*G* (kcal·mol^−1^)	▵*H* (kcal·mol^−1^)	−*T*▵*S* (kcal·mol^−1^)
EI_A_ + PEP	0.43 ± 0.12	−8.7 ± 0.2	3.6 ± 0.1	−12.3 ± 0.2
EI_A_^ΔEINα^ + PEP	0.037 ± 0.001	−10.1 ± 0.0	6.5 ± 0.1	−16.6 ± 0.1
EIC + PEP	260 ± 80	−4.9 ± 0.2	1.9 ± 0.4	−6.8 ± 0.4
Hinge motion		1.5 ± 0.2	−2.9 ± 0.2	4.3 ± 0.3
Swivel motion		−5.2 ± 0.2	4.6 ± 0.5	−9.8 ± 0.5
EI_A_(G356S) + PEP	14 ± 0.9	−6.6 ± 0.1	12.0 ± 0.2	−18.6 ± 0.2
EI_A_^ΔEINα^(G356S) + PEP	1.3 ± 0.1	−8.0 ± 0.1	14.5 ± 0.0	−22.5 ± 0.1
EIC(G356S) + PEP	310 ± 100	−4.7 ± 0.2	2.9 ± 0.4	−7.6 ± 0.4
Hinge motion		1.4 ± 0.1	−2.5 ± 0.2	3.9 ± 0.2
Swivel motion		−3.3 ± 0.2	11.6 ± 0.5	−14.9 ± 0.5

The hinge motion separates EINαβ and EINα within EIN and exposes hydrophobic interfacial residues between the subdomains, resulting in unfavorable entropic cost (Table 1). The energetic penalty of the hinge motion is largely compensated by the association between EINαβ and EIC accompanying the swivel motion, which buries wide hydrophobic interaction surfaces 7. The enthalpic and entropic changes upon the hinge motion were very similar between EI_A_ and EI_A_(G356S), indicating that the G356S mutation had little impact on the hinge motion (Table 1). The G356S mutation, however, profoundly influenced the thermodynamics of the association between EINαβ and EIC. EI_A_(G356S) exhibited much weaker domain association that was attributed to high enthalpic cost outweighing entropic gains from the association. The origin of large changes in enthalpy and entropy in EI_A_(G356S) is not clear, but the large difference in entropic contributions may reflect a less compact dimeric state of EI_A_(G356S).

### Interfaces for the association between EINαβ and EIC

**Figure 5:**
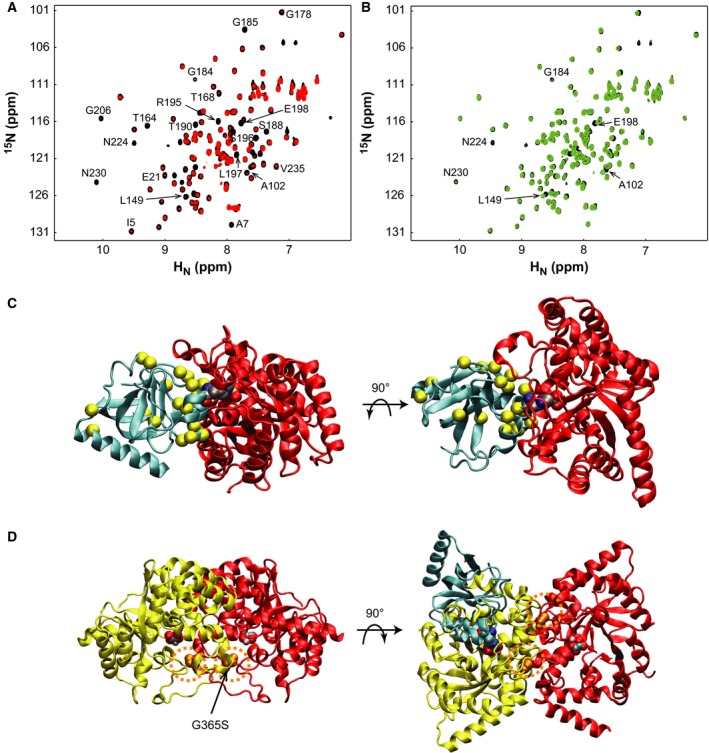
The ^1^H‐^15^N HSQC spectra of 0.3 mm ^15^N‐EINαβ_A_ (black) in the presence of 10 mm PEP titrated with (A) 0.45 mm unlabeled EIC (red) and (B) 0.45 mm unlabeled EIC(G356S) (blue). Residues of EINαβ_A_ that exhibited chemical shift changes or line broadening upon the titration are annotated by the residue types and numbers in the spectra. Side and top views (PDB code 2hwg) of (C) EINαβ associated with EIC and (D) the EIC dimer as a cartoon diagram. EINαβ is colored in cyan and EIC in red. EINαβ residues with line broadening are shown as yellow spheres. Individual subunits of the EIC dimer are colored in yellow and red, and Gly356 residues are shown as space‐filling models enclosed by dashed circles in orange in (D). EINαβ in cyan is added on the right panel to illustrate that the interaction surface of EIC for EINαβ is distant from the dimerization interface. The active site phospho‐His189 and oxalate bound on EIC are shown as space‐filling models as a visual guidance.

**Figure 6:**
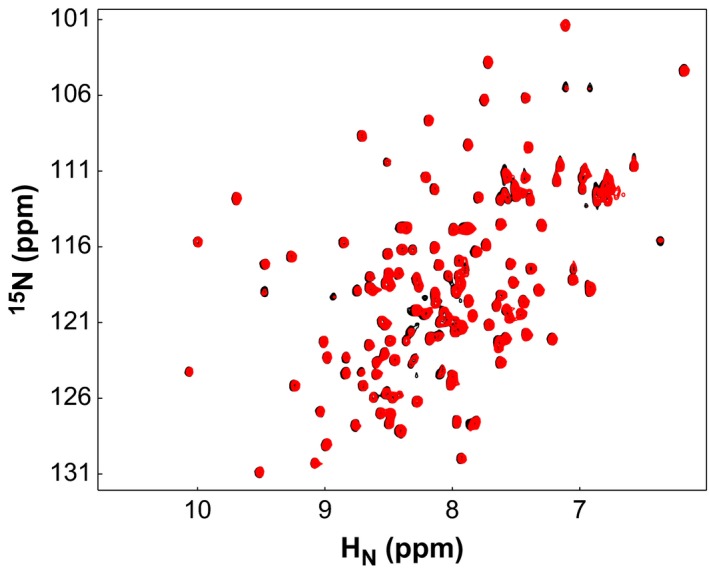
The ^1^H‐^15^N HSQC spectra of 0.3 mm ^15^N‐EINαβ_A_ (black) in the presence of 10 mm PEP titrated with 0.45 mm unlabeled EIC(G356S) (red).

We further investigated the impact of the G356S mutation on the interface ^15^N‐EINαβ for EIC by NMR titration. We monitored the ^1^H‐^15^N HSQC spectra of ^15^N‐EINαβ titrating with EIC or EIC(G356S). Neither of the titration experiments showed any change in the absence of PEP, indicating that EINαβ did not interact with EIC without PEP. This is consistent with previous data that EI adopts predominantly an open state in the absence of PEP 5. When 0.3 mm ^15^N‐EINαβ was complexed with 0.45 mm EIC in the presence of 10 mm PEP, several residues exhibited severe line broadening (Fig. 5A). Residues with the largest line broadening were I5, A7, A102, L149, T164, T168, G178, G184, G185, S188, T190, R195–E198, G206, and N230, and most of them were located at the interaction surface for EIC (Fig. 5C). On the contrary, a similar titration of 0.3 mm ^15^N‐EINαβ with 0.45 mm EIC(G356S) in the presence of PEP showed little changes, which is consistent with the weaker interaction between EIC and EINαβ of EI_A_(G356S) (Fig. 6). Increasing the concentration of EIC(G356S) up to 0.9 mm resulted in modest changes for a few residues that exhibited line broadening as well in the titration with EIC (Fig. 5B). Thus, EINαβ employs similar binding interfaces for both EIC(G365S) and EIC in the presence of PEP, albeit much weaker affinity for EIC(G365S).

The dimer interface of EI is mainly comprised of β3α3 loop (333–366) and β6α6 loop (453–477) of EIC. We suppose that the PEP binding, EI dimerization, and domain association events are connected by an intricate signaling network that is allosterically regulated by the β3α3 loop. The β3α3 loop not only constitutes the dimer interface, but also contains Arg358 that interacts with the phosphoryl group of PEP, propagating conformational changes between the dimerization interface and the PEP binding site (Fig. 5D). Initial PEP binding to EI likely adjusts the β3α3 loop conformation to provide optimal dimerization interfaces. A compact EI dimer, once formed, suppresses conformational dynamics prevailing in EIC to facilitate the EIC‐EINαβ domain association, as was demonstrated by NMR relaxation dispersion 20, 21. The sequential signaling cascade from PEP binding to compact dimerization to domain association triggers the overall open‐to‐closed conformational transition. When the G356S mutation is introduced to the β3α3 loop, the former signaling pathway from PEP binding to compact EI dimerization was not perturbed, but the subsequent signaling from compact dimerization to facilitated domain association was compromised. It is remarkable that the large conformational transitions in EI are sensitive to a single G356S mutation. We speculate that the hydroxyl side chain of Ser356 might form hydrogen bonds with neighboring Glu350 and Asn352 to distort the β3α3 loop conformation, which could be deleterious to the conformational transition. We note that the dynamic nature at the active site extends to the intermolecular interaction between EI and HPr, suggesting that the conformational plasticity may fine‐tune protein‐protein interactions in general 22.

In summary, we demonstrate that the dimerization of EI facilitates the conformational transitions required for the autophosphorylation reaction in an allosteric manner. The loop β3α3 of EIC is in the center for the allosteric regulation between PEP binding, compact dimerization, and conformational transitions of EI. A single G356S mutation in the loop β3α3 was enough to perturb the communication between the dimerization and the conformational transition, leading to a defect in EI autophosphorylation. The mechanistic link between protein dimerization and allosteric regulation may be general in other multidomain multimeric enzymes 23, 24.

## Author contributions

YJY and JYS conceived and designed the experiments; YJY and IK performed the experiments; KOL, YJY, IK, and JYS analyzed the data; and KOL, YJY, and JYS wrote the manuscript.
